# Photobiomodulation plus Adipose-derived Stem Cells Improve Healing of Ischemic Infected Wounds in Type 2 Diabetic Rats

**DOI:** 10.1038/s41598-020-58099-z

**Published:** 2020-01-27

**Authors:** Ali Moradi, Fatemeh Zare, Atarodsadat Mostafavinia, Sobhan Safaju, Amirhossein Shahbazi, Malihe Habibi, Mohammad-Amin Abdollahifar, Seyed Mahmoud Hashemi, Abdollah Amini, Seyed Kamran Ghoreishi, Sufan Chien, Michael R. Hamblin, Reza Kouhkheil, Mohammad Bayat

**Affiliations:** 1 Department of Biology and Anatomical Sciences, School of Medicine, Shahid Beheshti University of Medical Sciences, Tehran, Iran; 2 Department of Anatomy, Faculty of Medicine, Tehran Medical Sciences, Islamic Azad university, Tehran, Iran; 3 Department of Microbiology, School of Medicine, Shahid Beheshti University of Medical Sciences, Tehran, Iran; 4 Department of Immunology, School of Medicine, Shahid Beheshti University of Medical Sciences, Tehran, Iran; 5 Urogenital Stem Cell Research Center, Shahid Beheshti University of Medical Sciences, Tehran, Iran; 6 Department of Statistics, Qom University, Qom, Iran; 7 Price Institute of Surgical Research, University of Louisville and Noveratech LLC of Louisville, Louisville, KY USA; 8 Wellman Center for Photomedicine, Massachusetts General Hospital, Harvard Medical School, Boston, MA USA; 9 Department of Anatomical Sciences, Zanjan University of Medical Sciences, Zanjan, Iran

**Keywords:** Biophysics, Microbiology, Stem cells

## Abstract

In this study, we sought to investigate the impact of photobiomodulation and adipose-derived stem cells (ADS), alone and in combination, on the maturation step of wound healing in an ischemic infected delayed healing wound model in rats with type 2 diabetes mellitus (DM2). We randomly divided 24 adult male rats into 4 groups (n = 6 per group). DM2 plus an ischemic delayed healing wound were induced in all rats. The wounds were infected with methicillin-resistant *Staphylococcus aureus*. Group 1 was the control (placebo) group. Group 2 received only photobiomodulation (890 nm, 80 Hz, 0.324 J/cm^2^, and 0.001 W/cm^2^). Group 3 received only the allograft ADS. Group 4 received allograft ADS followed by photobiomodulation. On days 0, 4, 8, 12, and 16, we performed microbiological examination (colony forming units, [CFU]), wound area measurement, wound closure rate, wound strength, and histological and stereological examinations. The results indicated that at day 16, there was significantly decreased CFU (Analysis of variance, p = 0.001) in the photobiomodulation + ADS (0.0 ± 0.0), ADS (1350 ± 212), and photobiomodulation (0.0 ± 0.0) groups compared with the control group (27250 ± 1284). There was significantly decreased wound area (Analysis of variance, p = 0.000) in the photobiomodulation + ADS (7.4 ± 1.4 mm^2^), ADS (11 ± 2.2 mm^2^), and photobiomodulation (11.4 ± 1.4 mm^2^) groups compared with the control group (25.2 ± 1.7). There was a significantly increased tensiometeric property (stress maximal load, Analysis of variance, p = 0.000) in the photobiomodulation + ADS (0.99 ± 0.06 N/cm^2^), ADS (0.51 ± 0.12 N/cm^2^), and photobiomodulation (0.35 ± 0.15 N/cm^2^) groups compared with the control group (0.18 ± 0.04). There was a significantly modulated inflammatory response in (Analysis of variance, p = 0.049) in the photobiomodulation + ADS (337 ± 96), ADS (1175 ± 640), and photobiomodulation (69 ± 54) treatments compared to control group (7321 ± 4099). Photobiomodulation + ADS gave significantly better improvements in CFU, wound area, and wound strength compared to photobiomodulation or ADS alone. Photobiomodulation, ADS, and their combination significantly hastened healing in ischemic methicillin-resistant *Staphylococcus aureus* infected delayed healing wounds in rats with DM2. Combined application of photobiomodulation plus ADS demonstrated an additive effect.

## Introduction

Type 2 diabetes mellitus (DM2) is an important worldwide health problem with a high prevalence in the United States^1^. It is anticipated that the number of adults who suffer from DM2 will increase^2^. More than 100 million adults in the United States currently suffer from DM or pre-DM. It has been reported that 90–95% of patients with DM in the United States have DM2 and only 5% are recognized as having type 1 DM (DM1)^2^.

The repair of injured skin (skin wound healing) is a physiological reaction towards interruption of skin integrity that needs coordinated regulation of different cells and growth factors. This complex course of healing is susceptible to imbalance by DM and the consequences of ischemia^2^. DM2 can adversely affect each step of healing and lead to reduced numbers of blood vessels and deficient angiogenesis^3^, ischemia^4^, weak skin injury repair, formation of diabetic foot ulcers (DFU), and limb amputation^5^. The metabolic aberrations of DM from overproduction of mitochondrial superoxide in endothelial cells affect important pathways that result in complications for wound healing. Robust production of intracellular reactive oxygen species leads to damaged angiogenesis and ischemia^6^. DFUs are present in 15% of diabetics, from which approximately 14–24% will eventually undergo limb amputations. The death rate approaches 50–59% at 5 years after amputation^7^. DFUs, as an important type of chronic non-healing wound, are especially susceptible to infection due to the impaired host response in DM2 patients^8^.

Approximately 50% of all amputations are in patients with DM and are frequently due to bacterial infections of these DFUs that lead to gangrene. Among bacterial infections, *Staphylococcus aureus* is one of the most frequently diagnosed^9^. The prevalence of methicillin-resistant *Staphylococcus aureus* (MRSA) in infected DFUs is 15%–30%^10^. Excessive administration of inappropriate antibiotics for diabetic foot infections has resulted in concern about the levels of bacterial resistance^11^.

It is critical to accelerate the repair of injured wounds in diabetic patients, and this is assumed to be a crucial aim of DFU treatment^12^. Protective and therapeutic approaches to stimulate healing include the application of antibacterial substances, wet absorbent dressings, bioengineered gauzes, vacuum-assisted closure, Regranex PDGF gel, and unloading^13^. Nevertheless, none of these approaches has consistently demonstrated an advantage, and the cure of chronic wounds and DFUs is a prolonged, and difficult process^13^.

Stem cell therapy is evolving as a possible treatment for DFUs. Adult-derived stem cells are now employed in many commercially available products^2^. Engraftment of exogenously delivered adipose-derived stem cells (ADS) are recommended because of their ability to induce a favorable response as regenerative medicine for DFUs^14^. Growth factors secreted by ADSs can stimulate angiogenesis both in cell culture systems and animal models of ischemic tissue through paracrine signaling pathways^15^ and modification of the anti-inflammatory response within the injured tissue^16^. Therefore, ADSs may be used as a cell therapy for ischemic tissues in diabetics. Despite some success, two main barriers should be overcome in order to obtain convincing treatment benefits. Inadequate engraftment and limited viability of stem cells inside the injured tissue are primary problems. Hence, new tactics to maximize stem cell potential should be validated^2,17^. Autologous stem cell therapy approaches can be limited by the decreased function of stem cells obtained from patients with DM^18,19^.

Photobiomodulation is a tactic that can presumably overcome poor transplantation, viability, and DM-associated deficiencies of ADS. Photobiomodulation triggers healing, decreases pain, and lessens inflammation. It stimulates numerous transcription factors that enhance cell viability, augment cell proliferation and migration, and stimulate protein production^20^. Of interest, a few human studies (77 patients) have reported the therapeutic efficacy of photobiomodulation for DFUs in patients with DM2^21–24^. In a review paper, Beckmann *et al*. stated that most clinical trials reported the possible efficacy of photobiomodulation in repair of DFUs. However, a number of features in these trials limited the final evidence of the actual effectiveness of photobiomodulation. Beckmann *et al*. suggested that additional well-planned investigations would be necessary to prove the efficacy of photobiomodulation as standard wound care in humans^25^. An animal study conducted by Bayat group demonstrated the positive impact of photobiomodulation and metformin on the microbial flora and wound strength in wounds of rats with DM2^26^. The authors showed that photobiomodulation (890 nm, 80 Hz, 0.324 J/cm^2^) meaningfully hastened the repair of injured skin at the maturation step and significantly decreased colony forming units (CFUs) of bacteria in a non-genetic model of DM2 in rats^26^.

More than 100 recognized factors are involved in the insufficiency of wound healing in patients with DM2^27^. The use of a mixture of therapeutic agents and biological biostimulators could assist with treatment of non-healing wounds and show a synergistic effect to improve the success of healing in different wound models^28–30^.

In the present study we focused on the effects of photobiomodulation and ADS, alone and combined, on the maturation step of an ischemic, delayed healing, infected wound model in DM2 rats. The combined application of photobiomodulation and ADS could hasten the healing process and provide a treatment for severe cases of DFUs in patients with DM2.

## Results

### ADS marker expression

**Figure 1:**
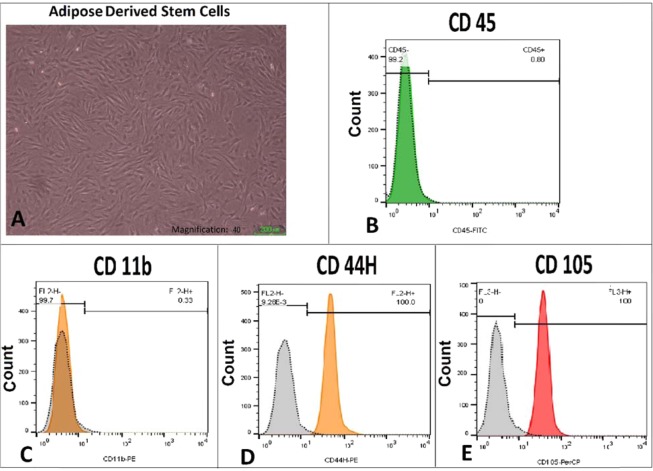
Passage-4 adipose-derived stem cells (ADS) grew as spindle-shaped, fibroblastic cell colonies (**A**). Immunophenotypes of these ADS cells showed that they expressed some clusters of differentiation (CD) 45 (**B**) and CD11b (**C**), and completely expressed CD 44H (**D**) and CD 105 (**E**).

The flow cytometry results showed that ADS expressed 0.33% CD11b and 0.8% CD45. In addition, 100% of ADS expressed CD44H and CD105 (Fig. 1).

### Observations of the rats

**Figure 2:**
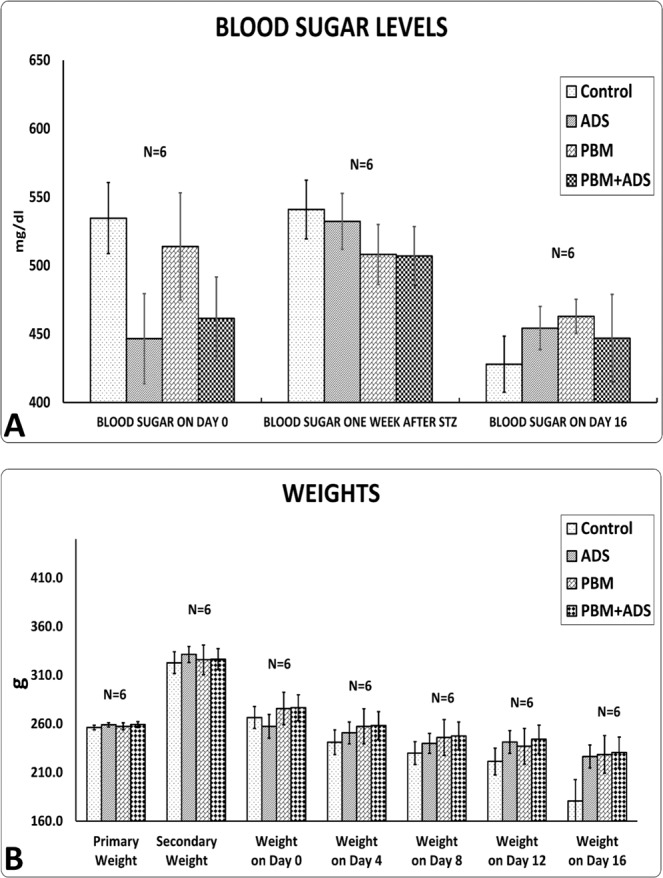
Following blood sugar levels (panel A) and body weight (panel B) changes of four studied groups by repeated measurements. Primary weight was measured at the beginning of feeding by fructose. Secondary weight was done at the end of feeding by fructose. ADS, adipose derived stem cell; PBM, photobiomodulation; STZ, Streptozotocin.

**Figure 3:**
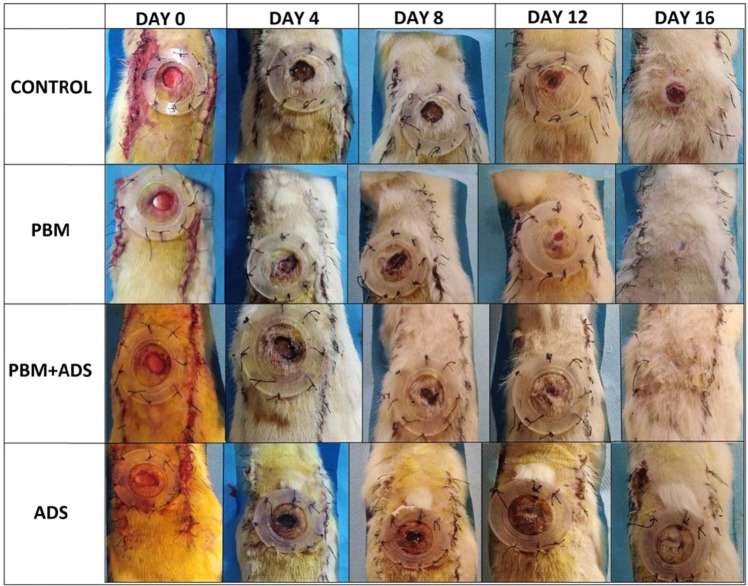
Progressive healing of the wounds in the four study groups over time. ADS, adipose-derived stem cell; PBM, photobiomodulation.

All rats developed clinical evidence of DM2. Initially, there was a significant increase in body weight after feeding with fructose followed by a significant increase in blood sugar levels and a significant decrease in body weight after treatment with Streptozotocin (Table 1). Figure 2 confirms the results of Table 1. In panel A of Fig. 2, repeated measurement analysis shows that after fructose treatment, the rats’ body weights increased (p = 0.000). After treatment with Streptozotocin, there was a significant decrease in body weight (p = 0.000) over time, such that the lowest weights of these rats were recorded on day 16. This weight loss occurred for all study groups. Panel B of Fig. 2 shows the change in blood sugar levels during the experiment. Although the blood sugar level increased one week after Streptozotocin administration, there was no significant difference in the blood sugar levels among the different groups. Figure 3 shows photographs from the wounds of rats in four studied groups, which represent progressive healing over time.

**Table 1:** Mean ± standard deviation (SD) of body weights and blood sugar levels of the four groups compared by paired student’s t-test.

GROUPS →	CONTROL	PBM	ADS	PBM + ADS
FACTORS↓				
BLOOD SUGAR AFTER STZ INJECTION (mg/dl)	509.6 ± 56.13	508.17 ± 53.61	507.7 ± 60.3	495.17 ± 51.7
BLOOD SUGAR ON DAY 16 (mg/dl)	513.6 ± 93.83	462.8 ± 123.06	454.4 ± 79.34	447 ± 78.57
INITIAL WEIGHT (g)	256.25 ± 6.41	257.5 ± 8.8	259.1 ± 6.58	259.29 ± 7.87
SECONDARY WEIGHT AFTER FRUCTOSE (g)	322.88 ± 31.55***	325.83 ± 37.2**	331.5 ± 24.55***	326.57 ± 28.31***
WEIGHT ON DAY 16 (g)	217 ± 30.54**	228.67 ± 47.27***	226.5 ± 28.87***	230.5 ± 38.88***

PBM, photobiomodulation; ADS, adipose-derived stem cells; STZ, Streptozotocin. Initial (primary) weight was measured at the beginning of the fructose feeding. The secondary weight was obtained at the end of the fructose feeding. **p < 0.01; ***p < 0.001.

### Microbial findings

#### Day 8

**Figure 4:**
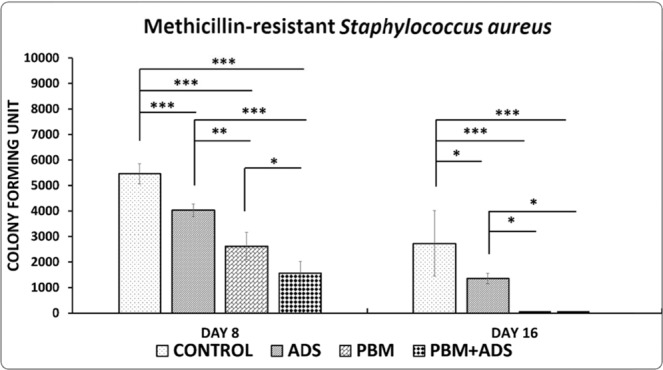
Mean ± SD of colony forming units of methicillin-resistant *Staphylococcus aureus* in wounds of the four study groups compared by Analysis of variance (ANOVA), and least significant difference (LSD) tests. *p < 0.05; **p < 0.01; ***p < 0.001.

All p-values were attributed to the least significant difference (LSD) test. Treatment with photobiomodulation + ADS, photobiomodulation, and ADS significantly decreased CFU of in the wounds compared with the control group (all, p = 0.000). In addition, photobiomodulation + ADS treatment was statistically better than treatment with only photobiomodulation (p = 0.012) and ADS alone (p = 0.002). Treatment with only photobiomodulation significantly decreased CFU in comparison with ADS treatment (p = 0.002, Fig. 4).

#### Day 16

There were no detectable CFU in the wounds treated with the photobiomodulation + ADS and only photobiomodulation. There were significant decreases in CFU in the photobiomodulation + ADS, photobiomodulation, and ADS groups compared with the control group (all, p = 0.000). Photobiomodulation + ADS and treatment with only photobiomodulation were significantly better than treatment with only ADS (both, p = 0.046, Fig. 4).

### Wound area measurement

#### Day 4

**Figure 5:**
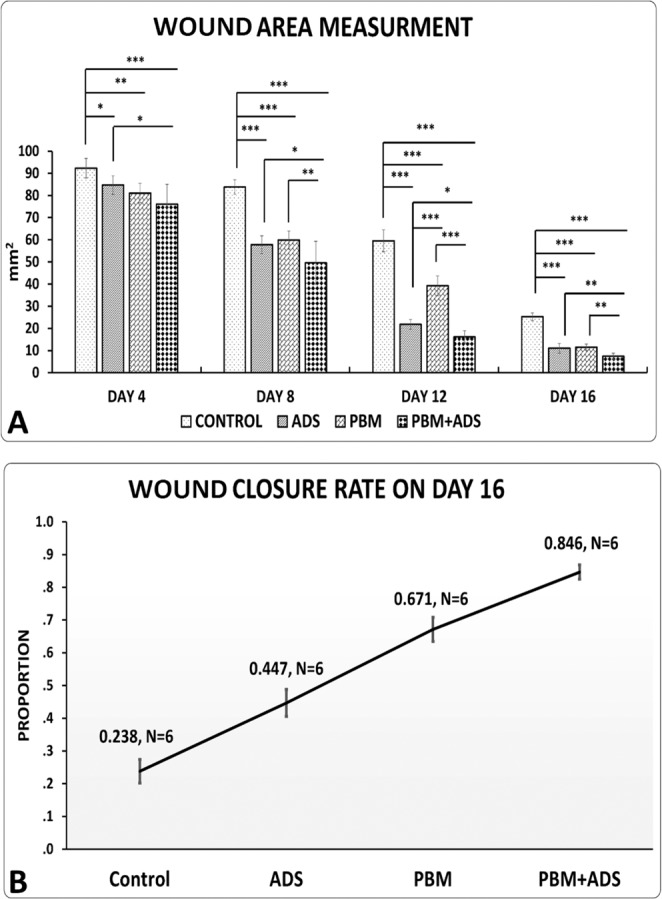
Mean ± SD of wound area measurement, (panel A) and wound closure rate (panel B) in wounds from the four study groups compared by ANOVA and LSD tests. *p < 0.05; **p < 0.01; ***p < 0.001.

Panel A of Fig. 5 shows the measurements of the wound areas of the study groups. Photobiomodulation + ADS, photobiomodulation, and ADS treatments significantly decreased the wound area compared with the control group (p = 0.000, p = 0.003, p = 0.028). At the same time, photobiomodulation + ADS treatment significantly decreased wound area compared with only ADS treatment (p = 0.014; Fig. 5, panel A).

#### Day 8

Photobiomodulation + ADS, photobiomodulation, and ADS treatments significantly decreased the wound area compared to the control group (all, p = 0.000). Simultaneously, photobiomodulation + ADS treatment significantly decreased the wound area compared to only the photobiomodulation (p = 0.006) and only ADS treatments (p = 0.019; Fig. 5, panel A).

#### Day 12

Photobiomodulation + ADS, photobiomodulation, and ADS treatments significantly decreased the wound areas compared to the control group (all p = 0.000). Both the photobiomodulation + ADS and ADS alone treatments significantly decreased wound area compared with photobiomodulation alone treatment (both p = 0.000). At the same time, photobiomodulation + ADS treatment significantly decreased the wound area compared with ADS treatment (p = 0.018). Treatment with only ADS significantly decreased the wound area compared with the photobiomodulation alone treatment (p = 0.000; Fig. 5, panel A).

#### Day 16

Photobiomodulation + ADS, photobiomodulation, and ADS treatments significantly decreased the wound area compared with the control group (all p = 0.000). Simultaneously, photobiomodulation + ADS treatment significantly decreased wound area compared to the photobiomodulation alone (p = 0.001) and ADS alone (p = 0.003) treatments (Fig. 5, panel A).

### Wound closure rate on day 16

Panel B of Fig. 5 shows the wound closure rates on day 16. The corresponding chi-square statistic for model fit was 0.118 with a p-value of 0.943, which represented an excellent fit of the logistic model. Based on this model, the wound closure percentages are given in panel B of Fig. 5. This figure shows that the photobiomodulation + ADS group had the highest closure rate of 0.846, while the control group had the lowest closure rate of 0.238.

### Findings of tensiometerical properties, bending stiffness

**Figure 6:**
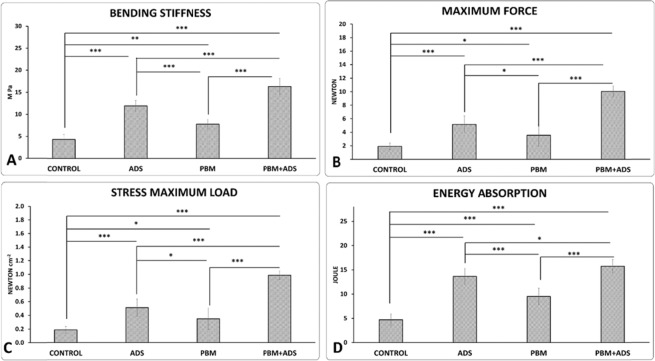
Mean ± SD of tensiometeric properties of bending stiffness (panel A), maximum force (panel B), stress maximum load (panel C), and energy absorption (panel D) of the wounds from the four study groups compared by ANOVA and LSD tests. *p < 0.05; **p < 0.01; ***p < 0.001.

Photobiomodulation + ADS, ADS, and photobiomodulation treatments showed significantly increased bending stiffness compared to the control group (p = 0.000, p = 0.000, p = 0.001). Concurrently, photobiomodulation + ADS treatment significantly increased bending stiffness to a greater extent than treatments with photobiomodulation alone and ADS alone (both p = 0.000). ADS treatment significantly increased bending stiffness in comparison with photobiomodulation treatment (p = 0.000; Fig. 6, panel A).

### Maximum force

Photobiomodulation + ADS, ADS, and photobiomodulation treatments significantly increased the maximum force compared to the control group (p = 0.000, p = 0.000, p = 0.034). Concurrently, photobiomodulation + ADS treatment significantly increased maximum force to a greater extent than the photobiomodulation alone and ADS alone treatments (both p = 0.000). ADS treatment significantly increased maximum force in comparison with the photobiomodulation treatment (p = 0.037; Fig. 6, panel B).

### Stress maximum load

There was significantly increased stress maximum load in the photobiomodulation + ADS (p = 0.000), ADS (p = 0.000), and photobiomodulation (p = 0.030) treatments compared with the control group. Concurrent administration of photobiomodulation + ADS significantly increased stress maximum load to a greater extent than photobiomodulation alone and ADS alone (both p = 0.000). ADS treatment significantly increased stress maximum load in comparison with photobiomodulation treatment (p = 0.032; Fig. 6, panel C).

### Energy absorption

Photobiomodulation + ADS, ADS, and photobiomodulation significantly increased energy absorption compared with the control group (all p = 0.000). Photobiomodulation + ADS treatment significantly increased energy absorption to a greater extent than treatment with only photobiomodulation (p = 0.000) and only ADS (p = 0.042). ADS treatment significantly increased energy absorption in comparison with photobiomodulation treatment (p = 0.000; Fig. 6, panel D).

### Stereologic findings

**Figure 7:**
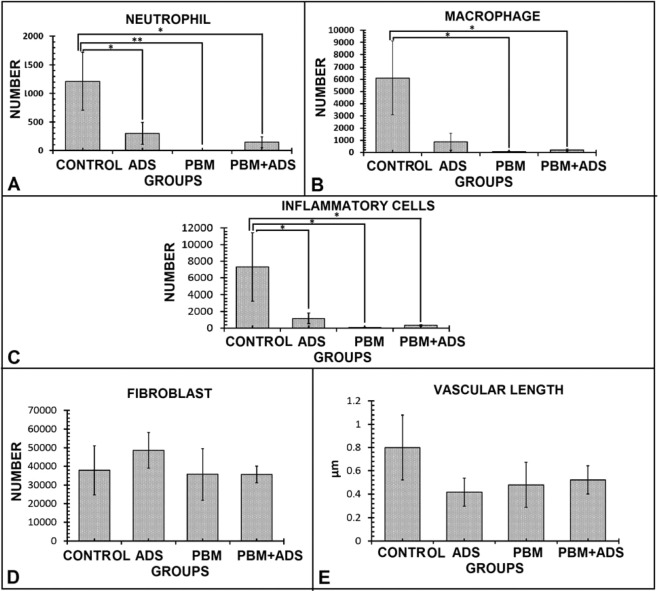
Mean ± standard error of the numbers of neutrophils (panel A), macrophages (panel B), inflammatory cells (panel C), fibroblasts (panel D), and the vascular length (panel E) of the wounds in the four study groups compared by ANOVA and LSD tests; *p < 0.05; **p < 0.01; ***p < 0.001.

**Figure 8:**
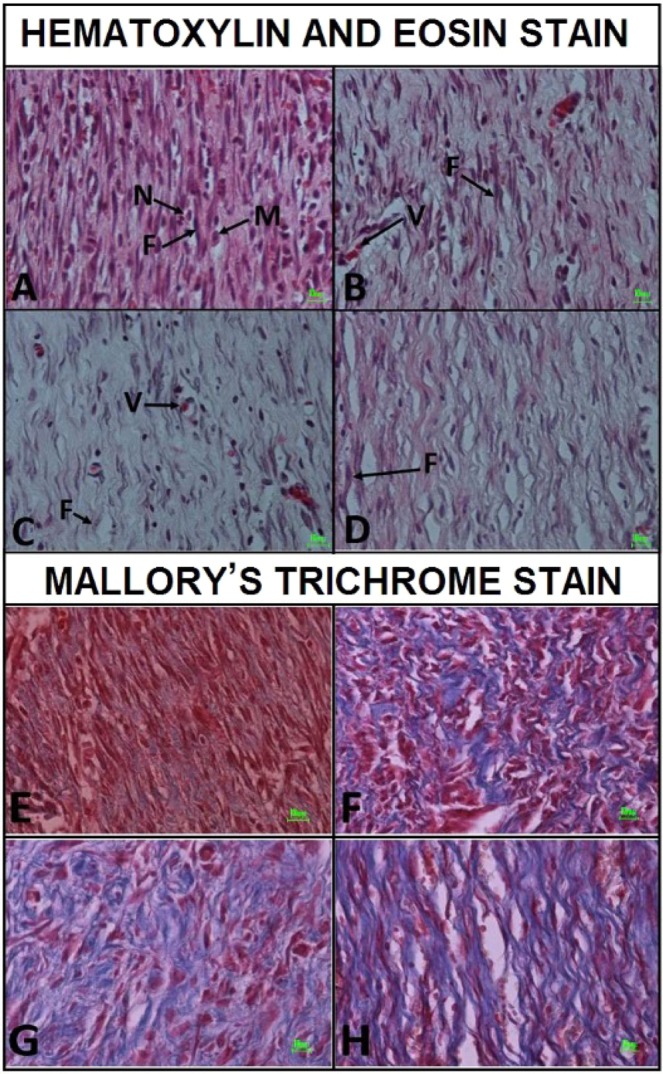
Representative histological images of the wound areas from the four study groups on day 16 according to Hematoxylin and Eosin staining and Mallory’s trichrome staining methods. Collagen fibers were detectable in blue color. F, fibroblasts; M, macrophages; N, neutrophils; V, blood vessels.

In terms of inflammatory cells, there were significantly better results (lower values) in the photobiomodulation + ADS, photobiomodulation, and ADS groups compared with the control group. There were no significant differences in fibroblast numbers and vascular length among the studied groups (Fig. 7). We observed significantly less neutrophil counts in the photobiomodulation (p = 0.006), photobiomodulation + ADS (p = 0.014), and ADS (p = 0.025) groups compared with the control group (Fig. 7, panel A). We observed a significant decrease in macrophage counts in the photobiomodulation (p = 0.038) and photobiomodulation + ADS (p = 0.041) groups compared with the control group (Fig. 7, panel B). Treatment with photobiomodulation, photobiomodulation + ADS, and ADS significantly decreased inflammatory cells (neutrophils plus macrophages) compared with the control group (p = 0.021, p = 0.026, p = 0.039; Fig. 7, panel C). Figure 8 shows the histological micrographs of the four study groups, which were stained with Hematoxylin and Eosin staining method.

### Results of mallory’s trichrome staining

In control group collagen fibers orientation were mixed, while in treatment groups were mostly horizontal (Fig. 8). In control group pattern of collagen fibers were mostly reticular and thin, whereas in treatment groups they were mostly fascicular and they seem thicker than collagen fibers of the control group (Fig. 8).

## Discussion

In this study, we investigated the impact of individual or combined administration of photobiomodulation and allograft ADS on the maturation step of wound healing in an ischemic delayed healing infected wound model in DM2 rats.

The results from this study indicated that all three treatment regimens significantly decreased CFU and the wound area, and significantly increased wound strength. Regarding CFU and wound area, co-treatment with photobiomodulation and ADS was significantly more effective than individual photobiomodulation or ADS treatments. Microbiological examination showed that photobiomodulation treatment was significantly better than ADS treatment. However, treatment with ADS was significantly better than photobiomodulation for the wound area measurement, and wound strength tests. Our assessment of inflammatory cells showed significantly better results (lower values) in the photobiomodulation + ADS, photobiomodulation, and ADS groups compared with the control group. There were no significant differences in fibroblast numbers and vascular length among the four study groups (Fig. 7). In current study the results of fibroblast count and Mallory’s trichrome staining were in the line of wound strength examination.

Complications with DFUs include an aberrant wound micro milieu, excessive inflammation, and angiogenesis maladies^31^. DFU occurs in the setting of ischemia, infection, neuropathy, and metabolic disorders. This results in poor wound healing and poor treatment options. In a review article, Lopes *et al*. have stated that existing proof points toward stem cell therapy as a persuasive cure for DFU^32^. Clinical and preclinical research studies have not provided any consensus regarding the optimum category of stem cell that should be used and there is no well-known optimal route or regimen for delivery of the stem cells. Differences within preclinical study designs suggest the need for an agreement regarding an optimum animal model that suggests translation to human studies. Variations in the stem cell category and source, route, and regimen for administration also confound easy interpretation and generalization of the results^32^. According to Lopes *et al*., there is an urgent need for additional experimentation to find a good animal model and persuasive regimens of stem cell therapy for treating severe cases of DFU. In the current study, for the first time, we have evaluated the combined effect of ADS plus photobiomodulation on a delayed healing wound model in rats. ADS are considered beneficial for use in regenerative medicine treatment regimens. Their chief benefit compared to mesenchymal stem cells extracted from other origins, such as the bone marrow, is their simple and reproducible extraction by relatively non-invasive methods. ADS also do not undergo immunological reactions and do not require matching of major histocompatibility antigen subtype for allogeneic engraftment^33,34^. ADS can differentiate into vascular endothelial cells^33^ and their engraftment stimulates new blood vessel formation and increases blood flow to the ischemic tissue in animal models^15,35^. It has been reported that growth factors released by ADS stimulate arteriogenesis in ischemic tissue by paracrine signaling^15^. A previous investigation from one of the authors of the current study has demonstrated that ADS immunomodulatory feature, brought it as an appropriate approach in treatment of some inflammatory diseases beyond the diabetic condition^36^. Recent investigations have shown a significant decrease in viable mesenchymal stem cell counts in animal models of skin injury and skull defects within the first 14 days of engraftment^37,38^.

One study demonstrated that some of the implanted ADS expressed endothelial markers in ischemic organs, but the hypoxic milieu of the ischemic organ by inducing cell death reduced the vascular incorporation rate of the ADS^39^. To improve this treatment, it is essential to improve stem cell function to enable their survival within ischemic tissue and have the capability to differentiate into vascular cells^40^. Approaches for promoting and increasing viability and engraftment of stem cells within ischemic tissue have been proposed and include transplantation mixed with a cytokine release system^40^, genetic modification of stem cells^41^, and the use of cell-implantation scaffolds^42^. While transplantation of stem cells plus the cytokine release system enhances stem cell viability, this approach presents difficulties for extensive medical use. A reproducible and organized delivery system should be established to extend the *in vivo* action of cytokines and prevent probable side effects^40^.

Guo and DiPietro, in their review article, emphasized the importance of wound strength in different medical situations^43^. Our study showed that treatment of wounds with ADS significantly increased wound strength compared to the control and photobiomodulation groups. This result showed the importance of ADS in treating nonhealing wounds such as diabetic wounds. However, we observed that combined administration of ADS and photobiomodulation demonstrated an additive effect.

Photobiomodulation has been extensively applied to enhance local circulation and improve injury repair by triggering new blood vessel formation in some non-diabetic animal models of ischemic tissues. Cury *et al*. have examined the impact of photobiomodulation (660 nm and 780 nm, 30 and 40 J/cm^2^) on three key mediators activated during new blood vessel formation in experimental models of random skin flaps. Tissues were collected from random skin flaps and they assessed the numbers of vessels, angiogenesis markers, and a tissue maturation marker. Cury *et al*. reported that photobiomodulation increased angiogenesis markers, and decreased tissue maturation markers. These phenomena varied according to energy density and depended on the wavelength^44^. In another study, Park *et al*. examined the effect of photobiomodulation on transplanted ADS in a mouse model of ischemic random skin flap and reported more cytokines released in the ADS + photobiomodulation group compared with the ADS group. ADS treatment improved tissue repair by endothelial cell differentiation and release of angiogenic growth factors. The ADS + photobiomodulation group showed better treatment efficiency in comparison with the ADS group, which was attributed to improved random skin flap viability, along with increased paracrine release. The enhanced paracrine release of angiogenic growth factors might be attributed to improve ADS viability by prevention of apoptosis^45^. Takhtfooladi *et al*. tested the impact of photobiomodulation (Al- Ga- In- P laser; 670 nm; 4 J/cm²; 40 mW/cm²) on ischemia-reperfusion of a skeletal muscle injury in rats^46^. They reported that photobiomodulation protected against the initial inflammatory response, prevented muscle atrophy and necrosis, and stimulated new blood vessel formation after the ischemia-reperfusion injury.

The effects of photobiomodulation plus conditioned medium of cultured human BMMSCs on infected wounds of diabetic rats have been studied by the Bayat group. In these animal studies, the researchers administered Streptozotocin to create a rat model of DM1. In the first study, Kouhkheil *et al*. reported the positive impacts of single or dual administration of photobiomodulation (890 nm, 80 Hz, 0.2 J/cm^2^) and/or hBMMSC-conditioned medium (4 injections) on CFU and wound strength of a MRSA infected wound model in DM1 rats^28^. In the second study, Fridoni *et al*. reported the beneficial effect of single or dual administration of photobiomodulation (890 nm, 80 Hz, 0.2 J/cm^2^) and/or conditioned medium from hBMMSC (four injections) on stereological parameters in an MRSA infected wound model in DM1 rats^29^.

To our knowledge, the effect of dual treatment with photobiomodulation plus ADS on the repair of infected wounds in DM2 animals (or humans) has not been reported. The present study aimed to assess the impacts of photobiomodulation plus ADS on microbial flora, wound area, wound strength, and histological and stereological parameters in DM2 rats. We found that the single administration of photobiomodulation or ADS, and the combined administration of photobiomodulation plus ADS significantly hastened wound repair in MRSA infected wounds of DM2 rats. Additionally, the combination of photobiomodulation and ADS provided an additive effect, which probably resulted from the enhanced survival of ADS due to the inhibitory effect of photobiomodulation on apoptosis and robust paracrine signaling of ADS^45,47^.

Lipoveski *et al*. conducted an *in vitro* study on the antibacterial property of blue light (415 nm, 100 mW, and 30, 60, 120 J/cm^2^), which showed the inhibitory effect of photobiomodulation on *Staphylococcus aureus* by reactive oxygen species induction^11^. It is possible that bactericidal effect of photobiomodulation treatment in our study could be attributed to the induction of reactive oxygen species. However, we used near-infrared light rather than blue light.

Different wavelengths of photobiomodulation have been tested on fibroblast survival and proliferation in a high glucose culture system. Cells irradiated in the red range of light (632.8 nm) showed a higher degree of cell viability and proliferation compared to cells subjected to photobiomodulation in the near-infrared range (830 nm). From these results, it might be concluded that diabetic cells could benefit from irradiation in the red range rather than the near-infrared range in terms of wound healing^48^. However, since near-infrared laser has a deeper penetration rate than red laser^49^, deeper wounds *in vivo* might require the use of near-infrared and infrared^48^ lasers such as the 890 nm laser used in the present study.

In the current study, stereological analysis supported our hypothesis that the treatments, particularly photobiomodulation plus ADS modulated the inflammatory response, simultaneously increased wound strength, and significantly decreased the wound area and CFU. Bayat and Chien have reported the positive effects of combined application of ADS and photobiomodulation in some ischemic tissues. They stressed that these results might support new healing attitudes for a cure and resolution of delayed healing of DFU in patients. They hypothesized that an ideal combination of mesenchymal stem cells and photobiomodulation would accelerate the wound healing process. This approach could modulate the immune system in diabetic patients who have delayed healing and infected DFU. The combined application of ADS and photobiomodulation would be significant, not only for advancing the development of a new treatment for delayed healing and infected DFUs in diabetic patients, but it would also provide new findings about modulating the inflammatory response in DFUs^50^.

However, more specific studies are necessary to understand the exact role of inflammation in the healing process. In order to investigate the effects of photobiomodulation and ADS on the promotion of M2 polarization *in vivo*, CD206/CD68 immunofluorescence staining should be performed on wound tissue sections and oxidative stress levels should be evaluated.

**Figure 9:**
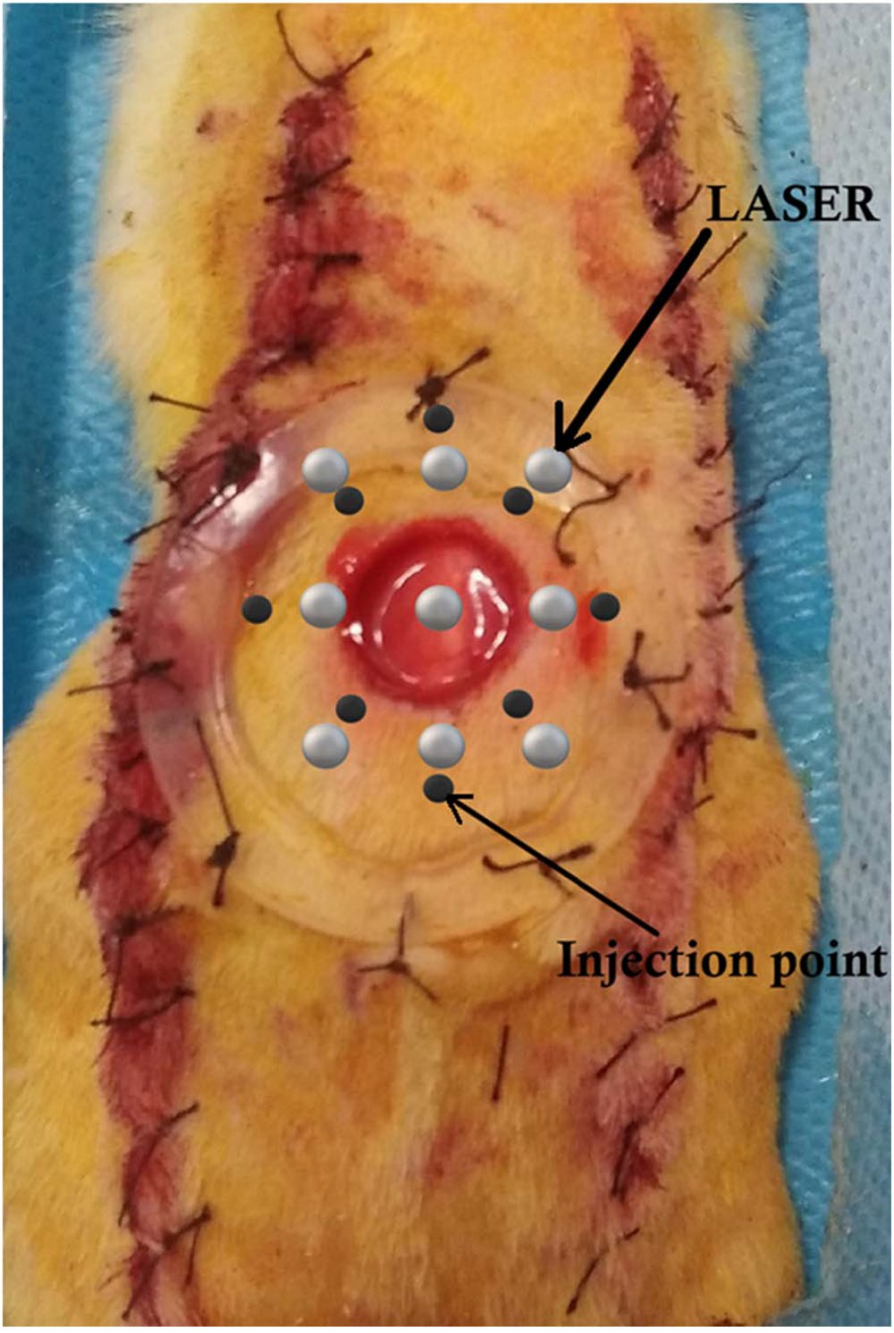
The wound, laser target sites, and adipose-derived stem cell injection points.

According to the literature, the bipedicle skin flap is an established model of ischemia^51–53^. In the current experiment, for the first time, we initially created a bipedicle skin flap on the back of each rat followed by an excisional wound in the flap. Finally, we inserted a silicone ring around the wound (Fig. 9). This approach could reduce the wound closure induced by the skin muscle^54^ and would allow the wound to predominantly rebuild by re-epithelialization and granulation tissue formation, which resemble the wound healing process in humans. Our pilot study has shown that this wound (ring + ischemic) model heals slower compared to a non-ischemic wound without any ring in healthy animals. Ischemia, neuropathy, and infection are three pathological components that lead to diabetic foot complications. They frequently occur together as an etiologic triad^55^. In the current experiment, we generated an ischemic delayed healing skin injury that was infected with MRSA to simulate the worst cases of DFU in an animal model.

## Conclusion

The application of photobiomodulation and ADS alone or in combination significantly hastened wound healing in an ischemic delayed healing MRSA infected wound model in rats with DM2. The combined use of photobiomodulation and ADS demonstrated an additive effect. We suggest that photobiomodulation combined with ADS could be investigated in human translational studies to reduce inflammation and infection, and promote healing. The details of the cellular and molecular mechanisms that concern the combined effect of photobiomodulation and ADS on the repair of ischemic infected wounds in diabetic rats should be elucidated by further research.

## Materials and Methods

### Animals and study design

We randomly divided 24, 3-month old male Wistar rats into four groups of six animals each. DM2 was induced in all of the rats. Next, we constructed an ischemic, delayed healing, infected wound model in all of the rats. Group 1 was the untreated control (placebo) group. Group 2 received photobiomodulation alone. In Group 3, allograft ADS were transplanted, and in Group 4, allograft ADS were transplanted followed by photobiomodulation administration. On days 0, 4, 8, 12, and 16, microbiological examination, wound area measurement, wound closure rate, and wound strength were assessed. In this study, day 4 was the inflammation step, day 8 was the proliferation step, and days 12 and 16 were considered to be the early and late (respectively) remodeling steps of wound healing. The IACUC of the National Institute for Medical Research Development (NIMAD), Tehran, Iran (file no: IR.NIMAD.REC.1397.256) and Research Department of the School of Medicine, Shahid Beheshti University of Medical Sciences, Tehran, Iran (IR.SBMU.MSP.REC.1398.295) approved this study. Handling and working with the rats were conducted in accordance with guidelines of the NIMAD and the Guide for the Care and Use of Laboratory Animals (8th edition).

### Induction of type 2 diabetes mellitus

Initially, the animals were fed with 10% fructose (Biobasic, Canada) in drinking water (instead of pure water) plus standard chow pellets for rats for 14 days. Next, each animal received an injection of intraperitoneal injection of Streptozotocin^56^. After seven days, the rats’ blood sugar levels were measured. Animals with blood sugar levels higher than 250 mg/dl were considered to have DM2^26^.

### Clinical examinations

Body weights and blood sugar levels of rats were monitored throughout the experiment.

### Surgery

The rats were anesthetized by intramuscular (i.m.) injections of ketamine (50 mg/kg) and xylazine (5 mg/kg). The rats received ceftriaxone (50 mg/kg, i.m.) before the surgery, and again at 24 h and 48 h after surgery. A dorsal, bipedicle skin flap (10 × 3.5 cm) was induced deep into the skin and underlying skin muscle. One, 12 mm full thickness excisional round wound that included the skin muscle was produced in the midpoint region of the flap using a biopsy punch. A donut-shaped silicone skin holder was fixed around each skin defect with a 04 silk suture (Fig. 9). Prior to surgery, all rats received 20 mg/kg ibuprofen every 8–12 h, which was continued until five days after surgery.

### Inoculation of MRSA into the wounds and microbiological examination

We used the MRSA strain ATCC 25923. The procedure was fully described in our previous study^28,29^. A single colony of MRSA was inoculated into liquid medium to a final concentration of 2 × 10^8^ cells/mL. A 100 μl aliquot of the suspension that contained 2 × 10^7^ MRSA cells was topically inoculated onto each wound immediately after surgery. We obtained microbiological samples for routine microbiological analysis from the wounds on days 8 and 16. The numbers of bacteria per sample were counted as CFUs.

### Photobiomodulation

The wounds of rats in groups 3 and 4 were subjected to photobiomodulation (Mustang 2000, LO7 pen, Technica Co., Russia, Table 2) while the rats were sedated (Fig. 9)^26^. In the present probe, the region of the target tissue (including the wound and surrounding skin, Fig. 9) was larger than the pen’s spot size. Therefore, we applied sequential treatments to ensure that each unit area received a similar laser energy density. Photobiomodulation was done over nine distinct regions, including the wound area and surrounding normal skin, with the laser pen held perpendicular to the target tissue and at a distance of less than 5 mm per area. The probe was covered with a very thin sterile disposal cover to ensure that infection was not transmitted to the other animals as well as transmission of the laser without any disturbance. During laser radiation, the irradiated animals were sedated by half doses of the anesthetizing drugs. Photobiomodulation was continued once per day, for six days a week until day 16.

**Table 2:** Photobiomodulation parameters.

Parameters	Dose and unit
Peak power output	75 W
Average power	1.08 W
Power density	0.001 W/cm^2^
Wave length	890 nm
Bandwidth	+/− 10 nm
Pulse Frequency	80 Hz
Spot size	1 cm^2^
Pulsed duration	180 ns
Duration of exposure for each point	300 s
Energy density	0.324 J/cm^2^
Number of laser shootings in each session	9
Total energy densities at 1 and total sessions	2.916, 40.824 J/cm^2^

### Preparation of allograft ADS

The ADS were obtained from adipose tissue within the abdominal region of healthy adult rats as described previously^57,58^. About 3 cm^3^ of adipose tissue was manually minced and subjected to digestion with 0.1% collagenase type I solution for 25 min at 37 °C, and then centrifuged. The cell pellets were suspended in Dulbecco’s Modified Eagle’s Medium (DMEM) plus 20% fetal bovine serum and then seeded into T-75 flasks that contained DMEM, 20% fetal bovine serum, 100 U/ml penicillin, and 100 µg/ml streptomycin. The ADS were characterized for mesenchymal stem cell markers by flow cytometry, as reported previously^57,58^.

### ADS transplantation

At 24 h after surgery, we suspended 1 × 10^6^ passage-4 ADS^47^ in 300 µl phosphate-buffered saline^45^. The rats received intradermal injections of this solution via an insulin syringe into eight sites around each wound (4–5 mm distance from the wound margins) (Fig. 9).

### Wound area measurement and wound closure rate

Photos of the wounds were taken with a digital camera on days 0, 4, 8, 12, and 16. The wound area (mm^2^) was computed and compared to day 0 using Image J-NIH (USA). The times of complete wound closure were recorded and reported as the wound closure rate^30^.

### Wound strength testing

On day 16, all of the rats were euthanized and we removed one, 5 × 50 mm sample from each wound. The samples were mounted in a material testing machine. The deformation rate was 10 mm/min. From the load-deformation curve, we calculated the tensiometric properties of maximum force (N), bending stiffness (MPa), stress maximum load (N/cm^2^), and energy absorption (J) of the samples^26^.

### Histological and stereological analyses

The samples from the euthanized rats were excised, prepared for light histological analyses, and serially sectioned into 5-μm sections. We stained ten sections with Hematoxylin and Eosin staining method and ten sections for Mallory’s trichrome staining method. In this study neutrophils, macrophages, fibroblasts, and vascular length were examined by stereological methods in H&E slides, and collagen fibers were examined semi descriptively in Mallory’s trichrome staining slides^59^

### Estimation of the cell numbers

The physical dissector method was used to determine the numerical density (Nv) of the neutrophils, macrophages, and fibroblasts as follows:

$${\rm{Nv}}=\Sigma Q/(h\times {\rm{a}}/{\rm{f}}\times \Sigma p)$$

where: **Nv**: Numerical density; **ΣQ**: Number of nuclei; **h**: Height of the dissector; **a/f**: Counting frame area; **Σp**: Number of counting frames in all fields.

$${\rm{N}}\,({\rm{total}}\,{\rm{of}}\,{\rm{cells}}\,{\rm{in}}\,{\rm{each}}\,{\rm{rat}})={\rm{Nv}}\times {\rm{V}}$$

where: **Nv:** Numerical density; **V:** Final total volume^60^

$${\rm{Estimation}}\,{\rm{of}}\,{\rm{vascular}}\,{\rm{length}}=2\Sigma {\rm{Q}}/(\Sigma {\rm{P}}\times {\rm{a}}/{\rm{f}})$$

where: **2ΣQ**: Total number of vessel profiles counted per rat skin; **ΣP**: Total number of vessel profiles counted per rat; **a/f**: Counting frame area^60^.

### Statistical analysis

Data are presented as mean ± standard deviation. We used the t-test for statistical analyses of body weight, one-way analysis of variance (ANOVA), and repeated measurement analysis. The LSD test was used for statistical analysis of body weight. ANOVA and the LSD tests were used for the microbiological examination results, wound area measurement, and wound strength tests. We analyzed the wound closure rate on day 16 for each group with the logistic regression model fit and chi-square tests. A p-value of < 0.05 was considered statistically significant.
